# Archaic Model Forms in Art Education and Eco Art-Therapy

**DOI:** 10.1192/j.eurpsy.2026.11457

**Published:** 2026-07-31

**Authors:** E. Chirila

**Affiliations:** Filiala Cluj, Uniunea Artistilor Platici Din Romania, Cluj-napoca, Romania

## Abstract

**Introduction:**

In order to counteract the effects of interethnic conflicts (which cause emotional distress to the young population) and to harmonize interethnic relations in a multicultural society, contemporary educational / art-therapeutical methods depend on the necessity to establish a sensitive relationship between the beneficiary’s cultural horizon and the means of education / art therapy. We experiment with art-specific ways to make interdisciplinary exchanges and cultural interferences using the universal language of visual arts along with intercultural elements and religious ecumenism.

**Objectives:**

It aims to develop the beneficiary through nonverbal communication capabilities, superior to verbal communication, using the universal quality of visual arts to transmit information beyond the linguistic boundaries. Social skills facilitate the social and professional integration of children and adolescents belonging to ethnic groups living together, including those with disabilities. With incursions into the history of universal, Romanian and Hungarian arts, we find the common elements of culture and arts.

**Methods:**

The person is followed as a whole, scientific data is used to model the organization of the inner life. Ecological / existential factors are analyzed, the relationship with the surrounding community is established: we benefit from a more complete circulation and ecology of expressive energy because we can see how the mechanisms of change allow the holistic transformation of the beneficiary. Through arts pedagogy and art therapy, traditional crafts of the arts are reinvented on levels: crafts, handicrafts, design, arts and visual arts are transformed into therapeutic methods through the preparation of techniques that belong to contemporary postmodern arts: artistic ceramics, design, mixed and multimedia with related arts.

**Results:**

Experimenting,with visible educational/art therapeutic effects, of complex relationships: shape of the human body – shape of man-made objects (in general) and the creation of personal shapes (of the beneficiaries). conduct to harmonize interethnic relations in a multicultural camp.

**Image 1:**
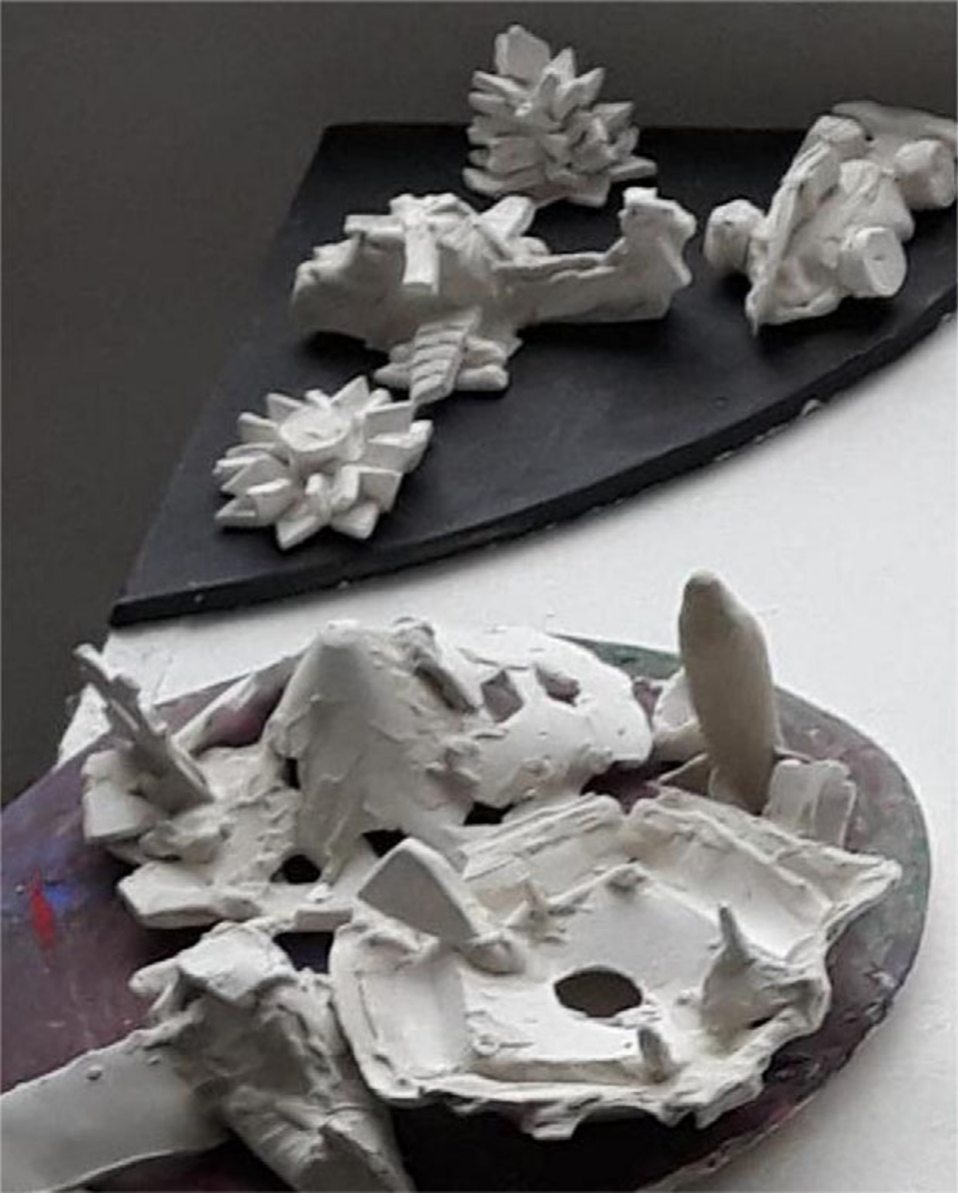

**Image 2:**
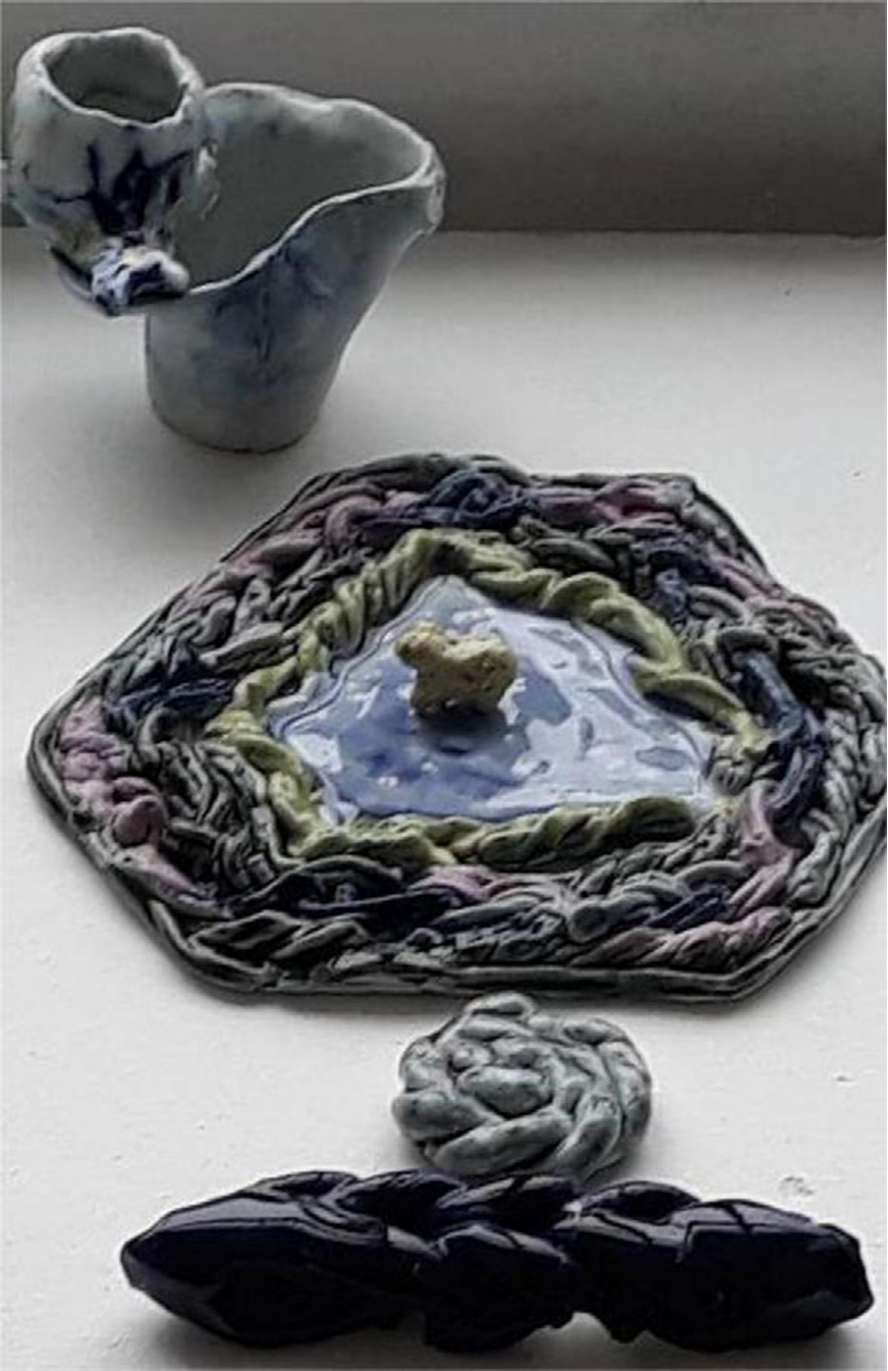

**Image 3:**
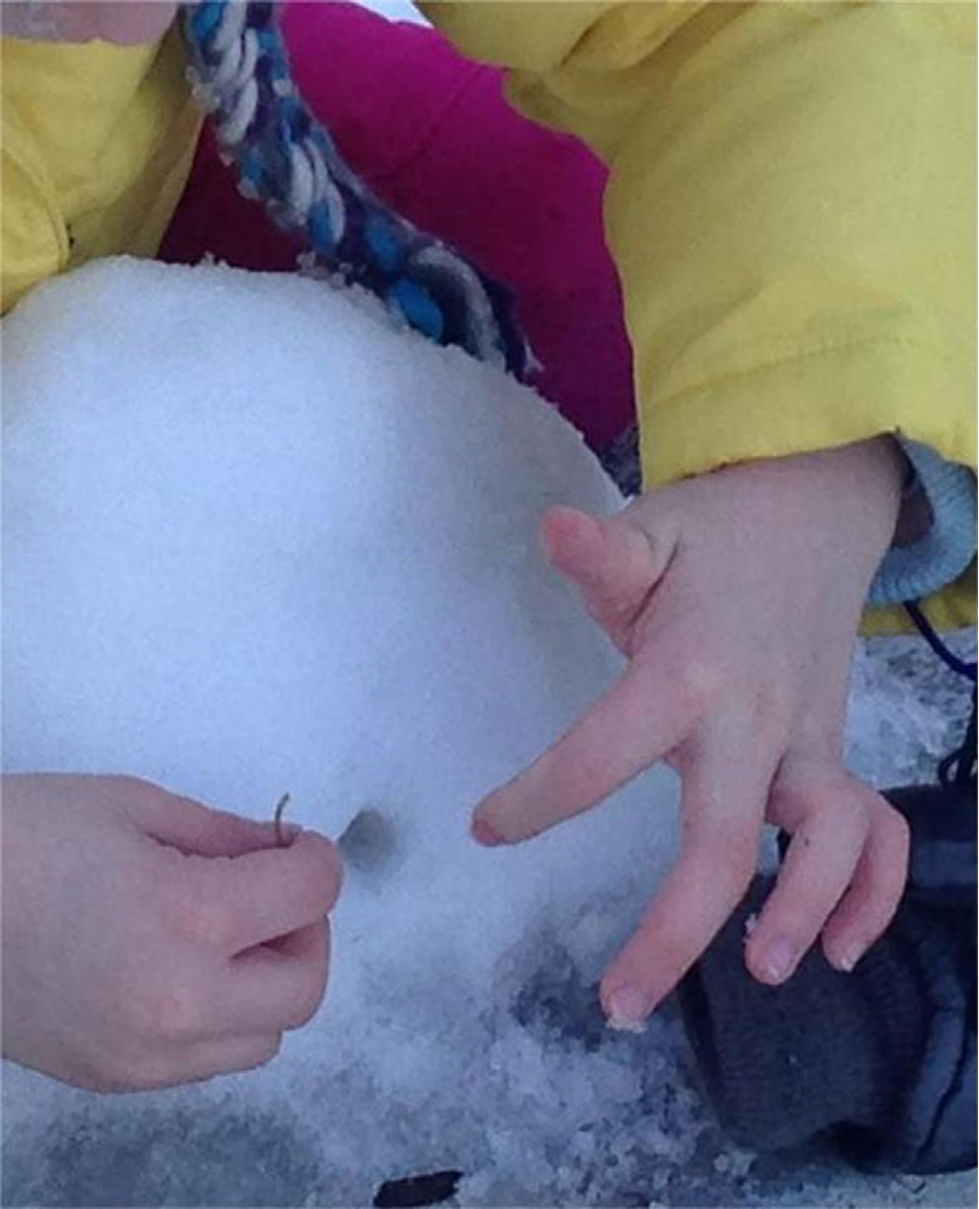

**Conclusions:**

The holistic approach to well-being aims to enable beneficiaries, who are in fragmented states, to achieve integrity, unity, harmony and balance, taking into account all the mental, emotional, physical and spiritual dimensions of human nature. Developing and maintaining social skills will be the conditions for increased adaptability and the development of personal transformation capacities.

**Disclosure of Interest:**

None Declared

